# Hepatitis C virus to hepatocellular carcinoma

**DOI:** 10.1186/1750-9378-7-2

**Published:** 2012-01-30

**Authors:** Shah Jahan, Usman A Ashfaq, Muhammad Qasim, Saba Khaliq, Muhammad Javed Saleem, Nadeem Afzal

**Affiliations:** 1 Department of Immunology, University of Health Sciences, Lahore, Pakistan; 2 Department of Bioinformatics and Biotechnology, Government college university (GCU), Faislabad, Pakistan; 3 Institute of Agriculture Sciences, University of Punjab, Lahore, Pakistan

## Abstract

Hepatitis C virus causes acute and chronic hepatitis and can lead to permanent liver damage and hepatocellular carcinoma (HCC) in a significant number of patients via oxidative stress, insulin resistance (IR), fibrosis, liver cirrhosis and HCV induced steatosis. HCV induced steatosis and oxidative stress causes steato-hepatitis and these pathways lead to liver injury or HCC in chronic HCV infection. Steatosis and oxidative stress crosstalk play an important role in liver damage in HCV infection. This Review illustrates viral and host factors which induce Oxidative stress, steatosis and leads toward HCC. It also expresses Molecular cascade which leads oxidative stress and steatosis to HCC.

## Introduction

Hepatitis C virus causes acute and chronic hepatitis [1] an can leads to HCC via oxidative stress, insulin resistance (IR), fibrosis, liver cirrhosis and HCV induced steatosis [2]. HCV is a major health problem, almost 350 million individuals are chronically HCV infected [3] and 10% of the Pakistani population is chronically infected with this viral pathogen [4]. Approximately 40-60% of HCV infected individuals leads to chronic liver disease [5], and prevalence of HCV associated HCC is higher in Pakistan as compare to the rest of world [6,7].

HCV is an enveloped positive-single stranded RNA virus 9.6 kb in length consisting of (Core, E1, E2 and possibly p7) proteins and nonstructural (NS2, NS3, NS4A, NS4B, NS5A and NS5B) viral proteins [8,9]. HCV Core is known as the inducer of steatosis, oxidative stress and HCC [10]. E1 and E2 are involved in virus attachment with the cells and are considered to be the first viral protein comes in contact with the cells [11]. p7 is possibly concerned with ion channel and virus assembly [12]. HCV has six genotypes and 52 subtypes mainly genotypes in the different region of the world are (1a, b, c, 2a, b, c, 3a, b, 4a, 5a, 6a). Due to absence of proofreading function of the RNA-dependent RNA-polymerase (NS5B), HCV has a high mutation rate and exists as genetically heterogeneous quasispecies in individual patients [13,14]. The genetic diversity is more than 30% in different genotypes and 20% in subtypes. Variations in amino acid sequence of different HCV genotypes cause difference in severity of pathogenesis. Recent studies have shown variable responses for interferon (IFN)-ribavirin combination therapy, oxidative stress/steatosis and insulin resistance due to the amino acid substitutions in the HCV Core region of different HCV genotypes.

HCV genotype 3a is mainly prevalent in Pakistan followed by 3b and 1a [15]. Moreover, a strong correlation between chronic HCV infection and HCC in Pakistan associated with genotype 3a has been observed which is not been observed in relation to any other genotype [6]. HCV genotype 3a is mostly involved in HCV induced steatosis [16]. HCV induced steatosis and oxidative stress causes steatohepatitis [17] and these pathways leads to liver injury or HCC in chronic HCV infection [10,18]. Steatosis and oxidative stress crosstalk play important role in liver damage in HCV infection [19].

Oxidative stress is a key contributor in HCV-induced pathogenesis (Tardif *et al*., 2005). Previous reports showed that HCV Core protein regulates gene expression and alter cell signaling pathways [20] leading to oxidative stress, liver steatosis and eventually HCC [21]. HCV Core protein also up regulates cyclooxygenase-2 (COX-2) expression in hepatocytes and causes oxidative stress leading to HCC [22,23]. Cellular genes inducible nitric oxide synthetase (iNOS) COX-2 and Vascular Epidermal Growth Factor (VEGF) regulate cellular growth and over expression of these genes has carcinogenic effects in liver cells [24]. COX-2 can induce angiogenesis growth factors via VEGF [25-27] and it has also been revealed that the over expression of COX-2 activates Akt by phosphorylation of Akt in human HCC [28]. Akt acts as a key signal mediator, which modulates cell survival and proliferation [29,30]. Different studies have shown the effect of HCV on these genes but effect of HCV genes on these cellular genes which are crucial for HCV pathogenesis has not been evaluated completely.

Steatosis, the most common cause of abnormal liver function, is a pathological condition in which simple triglyceride accumulation in hepatocytes cause hepatic steatosis that leads to HCC and other liver diseases (Tetri and Caldwll, 2003). In fatty acid synthesis pathway loss of adiponectin Receptor (Adipo R2) due to HCV activates of the enzyme acetyl-CoA carboxylase (ACC) [31] a rate-limiting enzyme for fatty acid synthesis. ACC catalyzes synthesis of malonyl CoA that is further converted into saturated fatty acids by the action of fatty acid synthase (FAS) [32]. In addition, the transcription factors such as sterol regulatory element binding protein (SREBP) and peroxisomal proliferator activator receptor-α (PPAR-α) are also the key player in both lipogenesis and fatty acid oxidation pathways. SREBP activates the entire gene involved in lipogenesis [33]. A major function of PPAR-α is to control fatty acid oxidation and activation and its deficiency results in defective fatty acid oxidation [34,35]. It has been demonstrated that HCV proteins may accumulate triglycerides in hepatocytes by modulating the fatty acid synthesis pathway which leads to steatosis [32]. However, the mechanism involved in the HCV induced steatosis is still not clear.

Apoptosis is fundamental process for the control and elimination of viral infections. The impact of apoptosis in chronic HCV infection is not well understood. It may be harmful by triggering liver fibrosis, or essential for viral elimination [36]. HCV proteins, particularly Core and NS5A protein have both pro- and anti-apoptotic effects. It is not known which HCV protein affects apoptosis and whether the infectious virions act pro- or anti-apoptotic. E1 and E2 are involved in virus attachment with the cells [37].

### Oxidative stress

Oxidative stress refers to the oxidation-reaction-dominant state of the living body induced by an imbalance between the oxidation reaction caused by reactive oxygen species (ROS) and the anti-oxidation reaction. Main ROS include superoxide (O2-), hydrogen peroxide (H2O2) and the hydroxyl radical (HO) [17]. Oxidative stress has emerged as a key contributor in the development and progression of many pathological conditions, including HCV-induced pathogenesis of liver [38]. Mainly in liver cells NO production which is induced by iNOS enzyme which is activated in inflammatory process.

### HCV induced oxidative stress

Oxidative stress is produced by inflammatory progressions that occur in hepatitis via immunological mechanisms. In addition, in HCV infectious disease, viral proteins have some functions in the induction of oxidative stress. In HCV infection, ROS are produced by NADPH oxidize and xanthine oxidase in neutrophils and macrophages [39]. It have been also demonstrated that oxidative stress plays an important role in CHC [40]. In the presence of hepatic steatosis, IR and increased levels of some cytokines, all of which are also stimulated by viral protein expression, oxidative stress is enhanced in HCV infection. In addition, HCV produced ROS also in hepatocytes in response to release of inflammatory cytokine from inflammatory cells [18,41]. A pathological relationship between oxidative stress and HCV infection is observed, the causes of oxidative stress in HCV infection are considered to include various factors such as mitochondrial damage, lipid accumulation and ER stress in the liver. Different studies revealed that viral proteins, mainly the HCV Core protein, cause oxidative stress. In this sense, inflammation in CHC is considered to be qualitatively different from inflammation observed in other types of hepatitis such as autoimmune hepatitis or hepatitis [17].

### HCV structural genes and oxidative stress

In addition to nucleocapsid formation, HCV Core protein also modulates gene transcription, cell proliferation, cell death, and cell signaling, interferes with lipid metabolism and suppresses host immune response [20]. Core is an important pathogenic determinant of HCV induced oxidative stress leading to liver steatosis, and HCC [21,42]. In addition, it is notable that in HCV infection the viral proteins themselves as well as inflammation due to hepatitis are regarded as a cause of oxidative stress [23]. HCV Core protein is known as the inducer of steatosis, oxidative stress and HCC [10]. HCV structural genes, particularly the Core protein, have different functions with respect to host cells [43] and are closely related to oxidative stress. Core protein is also able to up regulate ROS production by induced nitric oxide Synthetase (iNOS) which activates cyclooxygenase-2 (COX-2) expression in hepatocytes derived cells, providing a potential mechanism for oxidative stress leading to HCC [22,23]. The Core protein was found to have a variety of actions, including the induction of oxidative stress and accumulation of lipids, in cultured cells and transgenic mice [10,23] and an enhanced ROS production caused by damage of the mitochondrial electron transport system was observed in Core-protein-expressing cells [23]. Mitochondrial DNA, which has no protective proteins such as histone, is susceptible to damage by ROS in response to HCV and causes Apoptosis [44,45] and could also increase oxidative stress caused by damage of the electron transport system. Core is an important pathogenic determinant of HCV induced oxidative stress. It is also able to up regulate COX-2 expression in hepatocyte derived cells, providing a potential mechanism for hepatic fibrosis during chronic HCV infection [23].

### Cellular genes involved in HCV induced oxidative stress and HCC

#### iNOS

In an inflammation, iNOS is induced in macrophages and hepatocytes [46,47]. iNOS is related to a high-output pathway of oxidative stress and is responsible for various pathological processes [25]. In fact, iNOS synthesis correlates with intra hepatic viral load in CHC [48]. NO causes cellular damage upon its reaction to O2 and finally produced nitro tyrosine, which was observed in association to inflammation severity in CHC tissue [49] suggesting that the production of both NO and ROS increased. ROS and RNS are produced as defense factors against viral infection, but these. ROS and RNS also have cytotoxic effects. Over production of iNOS and COX-2 have carcinogenic effects achieved either directly or by producing mediator that regulate cellular growth [25-27]. Elevated NO production has been implicated as a cause of tissue damage by inflammation, thus contributing to liver tumor promotion [50]. NO has been shown to induce oxidative DNA damage and inhibit DNA repair [51]. The expression of both COX-2 and iNOS was significantly elevated in HCV-positive HCCs. These findings suggest that combined expression of iNOS and COX-2 may play an important role in the prognosis of HCV-positive HCC patients. Rahman *et al. *in 2001 examined COX-2 expression significantly correlated with iNOS expression playing an important role in prognosis of HCV-positive HCC patients that could be partially attributable to modulation of angiogenesis by COX-2 and a combined negative expression of iNOS and COX-2 had a significant impact on patient survival.

#### COX-2

Two isoforms of COXs have been identified, COX-1 and COX-2, both catalyzing the same enzymatic reaction. Despite the structural similarity, COX-1 and COX-2 are regulated and function differently [52]. COX-2 is not constitutively expressed, but is rapidly induced by both inflammatory and mitogenic stimuli resulting in increased Prostaglandins (PG) synthesis in neoplastic and inflamed tissues. PGs play a central role in inflammation, and COX is the key enzyme in the conversion of arachidonic acid to prostaglandins. COX-2 is induced by a variety of factors such as ROS, cytokines, growth factors and tumor promoters and has been connected to inflammation and the inhibition of apoptosis leading to carcinogenesis [53,54]. The expression of COX-2 in HCC was found to correlate with the levels of several key molecules implicated in carcinogenesis such as iNOS activate VEGF and p-Akt [55,56]. iNOS and COX-2 have carcinogenic effects achieved either directly or by producing mediator that regulate cellular growth [24]. COX-2 can induce angiogenesis growth factors via VEGF [25-27].

Upregulation of COX-2 has been detected in HCC [25,55,57]. COX-2 have carcinogenic effects [58,59] achieved either directly or by producing mediators that regulate cellular growth. COX-2 can induce angiogenesis via VEGF and PG production [25-27] and can also inhibit apoptosis by inducing the antiapoptotic factor Bcl-2 as well as activating antiapoptotic signaling through Akt/PKB. Phospho-Akt-Ser473 is also upregulated and causes the activation of COX-2 expression in liver cells. COX-2 plays an important role in VEGF-induced angiogenesis via p38 and JNK kinase activation pathways as VEGF-induced cell proliferation was significantly reduced when transfected with COX-2 siRNA (34.12 ± 5.81%) [60]. COX-2 expression in Huh-7 cell line was inhibited by selective COX-2 inhibitor (SC-58635) or COX-2 siRNA and dramatically suppressed the proliferation, migration, and differentiation *in vitro *and in vivo. An approximately 78% reduction of VEGF level has been found in COX-2 siRNA treated Huh-7 confirmed the role of COX-2 in induction of VEGF. Moreover, COX-2/PGE_2_/EP/VEGF pathway possibly also contributes to tumor angiogenesis in HCC [61]. COX-2-specific siRNAs were electroporated into SNU-387 cells and significant, sequence specific reductions in COX-2 expression, PGE2 production, and cell proliferation were observed (Park *et al*., 2006). Recent evidence indicates that COX-2 modulates angiogenesis either by augmenting the release of angiogenic peptides by tumor cells or by directly increasing the production of PGs [24,62].

#### PGE 2

PGs play a central role in inflammation, and COX is the key enzyme in the conversion of arachidonic acid to PG. The first step in the formation of PG is the liberation of arachidonic acid (AA) from membrane-bound phospholipids COXs [63,64]. It appears that inflammation-mediated induction of COX-2 may represent a pivotal step in hepatocarcinogenesis. Recent experimental evidence suggests that increased COX-2 expression and PG production may contribute to the development of HCC. Several recent studies indicate that the COX-2/PGE2 pathway is involved in HCC cell invasion [65,66]. Leng *et al. *in 2003 showed that COX-2 and PGE2 promote the growth of human HCC cells. In cultured HCC cells, over expression of COX-2 or treatment with PGE2 enhances VEGF production and this effect is blocked by inhibition of COX-2 [26]. Transfection of human HCC cell lines Hep3B and HepG2 with COX-2 expression vector or treatment with exogenous PGE2 induced phosphorylation of serine/threonine protein kinase B (Akt) and enhanced cell growth [28]. The observations that celecoxib inhibits the production of PGE2 in HCC cells and that COX-2 or PGE2 partial protects against celecoxib-induced apoptosis suggest the involvement of COX-2 inhibition in celecoxib-induced inhibition of HCC cell growth [28]. Therefore, in addition to hepatocarcinogenesis, COX-2-controlled PGE2 production signaling may also contribute to the pathogenesis of chronic hepatitis and cirrhosis.

#### p Akt

The expression of COX-2 in HCCs was found to correlate with the levels of several key molecules implicated in carcinogenesis, such as p-Akt [28], VEGF [26,27] and iNOS [25,28]. Leng *et al *(2003) showed a positive correlation between the expression of COX-2 and phosphorylated Akt/Protein kinase B (PKB) in human HCC, suggesting a potential role of Akt in COX-2-mediated hepatocarcinogenesis. The level of COX- 2 expression and Akt phosphorylation is positively correlated in cultured HCC cells and in human liver cancer tissues. Furthermore, inhibition of Akt activation by phosphatidylinositol 3-kinase (PI3-kinase) inhibitor LY294002 significantly decreased the viability of HCC cells [28]. These findings suggest an important role of Akt activation in COX-2-induced HCC cell survival. The observations that celecoxib treatment decreased the phosphorylation of Akt and that inhibition of Akt reduced HCC cell viability suggest that involvement of pAkt in HCC [28,30].

#### VEGF

Vascular Epidermal Growth Factor synthesis occurs via oxidative stress and calcium signaling induced by HCV gene expression. VEGF the most potent angiogenic factor for tumor angiogenesis is believed to be involved in COX-2-mediated angiogenesis [58,59]. COX-2 can induce angiogenesis via VEGF and PG production. Moreover, COX-2/PGE_2_/EP/VEGF pathway possibly also contributes to tumor angiogenesis in HCC (Zhao *et al*., 2007). There is also compelling evidence suggesting an important role of angiogenesis in COX-2-mediated hepatocarcinogenesis. COX-2 has been shown to induce angiogenesis via VEGF [62,67]. Two recent studies show that elevated COX-2 expression correlates with increased VEGF level and micro vascular density in human HCCs [26,27]. There is also evidence that the COX-2 inhibitor reduces the production of VEGF, a potent stimulator of angiogenesis in Huh-7 cells [26].

### Mechanism of oxidative stress leading to HCC

Oxidative stress has emerged as a key contributor in the development and progression of many pathological conditions, including HCV-induced pathogenesis of liver (Tardif *et al*., 2005). The expression of COX-2 in HCC was found to correlate with the levels of several key molecules implicated in carcinogenesis such as iNOS, VEGF and p-Akt [25,55]. COX-2 and iNOS have carcinogenic effects achieved either directly or by producing mediator that regulate cellular growth [24]. COX-2 can induce angiogenesis growth factors via VEGF (Rahman *et al*., 2001; Cheng *et al*., 2004; Tang *et al*., 2005). VEGF is also stimulated by a number of other inflammatory mediators including NO and certain cytokines. Previously, it has been shown that the over expression of COX-2 activates Akt in human HCC via a p13-kinase-dependent mechanism [28]. Akt acts as an important signal mediator, which regulates cell survival and proliferation [29,30]. High nitric oxide production has been concerned as a cause of tissue damage by inflammation, thus contributing to liver tumor promotion [50]. The expression of both COX-2 and iNOS was significantly elevated in HCV-positive HCCs; the level of COX-2 expression was significantly correlated with iNOS expression [25]. These findings suggest that combined expression of iNOS and COX-2 may play an important role in the prognosis of HCV-positive HCC patients. A few studies have revealed that COX-2 expression is correlated with VEGF expression in HCC [25-27]. COX-2/PGE_2_/EP/VEGF pathway possibly also contributes to tumor angiogenesis in HCC (Zhao *et al*., 2007). Expression of HCV sub genomic replicons or replicon RNA in human HCC cells increases COX-2 expression at the level of transcription through HCV-induced oxidative stress and subsequent activation of NF-kB (Cheng *et al*., 2004). The positive correlation between COX-2 and VEGF in the noncancerous liver tissues (Tang *et al*., 2005) suggests that COX-2 may also be involved in the angiogenesis in chronic liver disease through the VEGF pathway.

### HCV induced oxidative stress and steatosis lead to HCC

**Figure 1:**
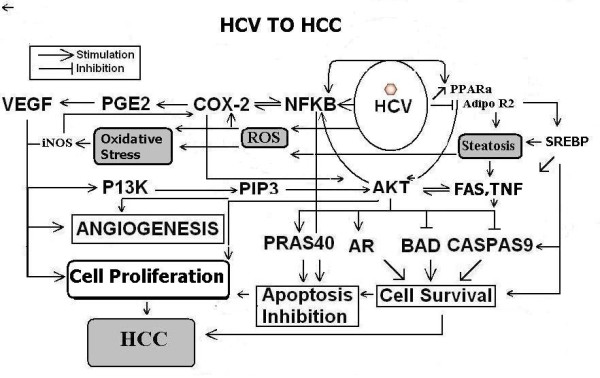
**Schematic representation of HCV to HCC**.

Oxidative stress and steatosis is supposed to play a pivotal role in the development of liver injury or HCC in chronic HCV infection (Figure 1) [10,18]. It has been reported that HCV genotype 3a is mostly involved in oxidative stress and HCV induced steatosis [16], which both contributes in the development of HCC [21,42,68]. Studies have shown the occurrence of oxidation stress and lipid peroxidation in CHC patients leads to HCC [69]. HCC is one of the most common causes of malignancy-related death in Africa and Asia (Koga, 2003). The role of oxidative stress in the progression of chronic hepatitis and hepatocarcinogenesis is greater in hepatitis C than in other types of hepatitis such as hepatitis B or autoimmune hepatitis. The additive effects of oxidative stress caused by the inflammatory process and that induced by HCV proteins may, furthermore, exert synergistic effects with alterations in intracellular signaling systems such as MAPK, which are also induced by HCV proteins. These synergistic effects may be responsible for rare characteristics, that is, the high incidence and multicentric nature of hepatocarcinogenesis in HCV infection. According to other proposed mechanism HCV Core and NS proteins may accumulate ROS which results in the peroxidation of membrane lipids and structural proteins, which are involved in the trafficking and secretion apparatuses, which block the VLDL secretion and causes mitochondrial dysfunction, DNA and cellular protein damage and further aggravate oxidative stress [42]. ROS production causes kupffer cells bursting which results in release of cytokines: TNFα, IL6 and IL8 [15]. TNFα down regulate the adiponectine thus, inducing IR and steatosis.

In human HCV genotype 3, which is most common in Pakistan, is more commonly associated with steatosis [70]. HCV induced Steatosis and oxidative stress is the most frequent cause of abnormal liver function. The Core protein was found to have a variety of actions, including the induction of oxidative stress and accumulation of lipids, in cultured cells and transgenic mice [10,23]. An increased ROS production and increased levels of intrahepatic peroxide lipids was observed in the Core gene transgenic mice as compare to control mice also show increased oxidative stress. Increased ROS production has been reported due to ß-oxidation in the mitochondria and peroxisomes or the metabolism of fatty acids by cytochrome P450 2E1 (CYP2E1) in microsomes is promoted under an excessive load of fatty acids (Day and James. 1998: Weltman *et al*., 1998). In HCV infection intrahepatic fat accumulation possibly increases ROS production as in HCV induced steatosis. Because hepatic steatosis in chronic hepatitis C was reported to be a cause for disease development (Czaja *et al*., 1998) increased oxidative stress related with hepatic steatosis is most probably involved in disease progression. Oxidative stress markers, 8-Hydroxy deoxyguanosine (8-OHdG) and peroxylipids were found to be decreased in PPAR-α KO Core gene transgenic mice which contributes to increased production of oxidative stress [10]. HCV Core protein down regulates the adiponectine receptor which further down regulates PPAR and up regulates JNK, AP-1 and cyclin D1 which results in, activation of cell growth signaling leads to HCC. On the other hand down regulation of adiponectine receptor also activates hepatic cells HSC due to which net collagen synthesis increases which lead to fibrosis and HCC.

### HCV and disease progression

HCV can leads to IR, fibrosis, liver cirrhosis and steatosis in a substantial number of patients [71-73]. Some studies showed that steatosis is more associated with fibrosis progression in HCV patients infected with genotype 3 i.e. patients with viral induced steatosis [74,75]. Other studies showed that metabolic steatosis is significantly associated with more sever fibrosis [76,77]. High level of TNF-α in CHC patient has been shown to induce IR in cultural cells and animal models [78,79]. Several studies reveal that IR is associated with non-alcoholic steatohepoatitis (NASH) and is also associated with a high risk of HCC that means IR increases the risk of chronic liver diseases and HCC. Moriya and colleagues showed that transgenic expression of Core protein leads to extensive steatosis, increased oxidative stress, mitochondrial injury and HCC in aging mice [21]. HCV induced steatosis and oxidative stress causes steatohepatitis [17] and these pathways leads to liver injury or HCC in chronic HCV infection [10,18]. Steatosis and oxidative stress crosstalk play important role in liver damage in HCV infection.

## Conclusion

The review describes viral and host factors which induce Oxidative stress, steatosis and leads toward HCC. Molecular cascade that leads to oxidative stress and steatosis to HCC has also been illustrated. In future, these biomarkers and therapeutic tartget can be used in the treatment of hepatocellular carcinoma.

## List of abbreviations

HCV, HCC, Oxidative stress, Steatosis, HCV pathogenesis Core, HCC

## Competing interests

All authors have no kind of institutional or financial competing interests.

## Authors' contributions

SJ, UAA designed and wrote the manuscript.SK, MQ, MJS and NA helped me in manuscript writing. All authors read and approved the final manuscript.

## Authors' information

Shah Jahan (PhD Molecular Biology), Usman Ali Ashfaq (PhD Molecular Biology),

Saba Khaliq (PhD Molecular Biology), Muhammad Qasim (PhD Molecular Biology)

Javed Saleem (PhD Molecular Biology), Nadeem Afzal (MSc Immunology)
